# A hybrid attention and dilated convolution framework for entity and relation extraction and mining

**DOI:** 10.1038/s41598-023-40474-1

**Published:** 2023-10-10

**Authors:** Yuxiang Shan, Hailiang Lu, Weidong Lou

**Affiliations:** China Tobacco Zhejiang Industrial Company Limited, Hangzhou, 311500 China

**Keywords:** Computer science, Information technology

## Abstract

Mining entity and relation from unstructured text is important for knowledge graph construction and expansion. Recent approaches have achieved promising performance while still suffering from inherent limitations, such as the computation efficiency and redundancy of relation prediction. In this paper, we propose a novel hybrid attention and dilated convolution network (HADNet), an end-to-end solution for entity and relation extraction and mining. HADNet designs a novel encoder architecture integrated with an attention mechanism, dilated convolutions, and gated unit to further improve computation efficiency, which achieves an effective global receptive field while considering local context. For the decoder, we decompose the task into three phases, relation prediction, entity recognition and relation determination. We evaluate our proposed model using two public real-world datasets that the experimental results demonstrate the effectiveness of the proposed model.

## Introduction

Relation extraction and mining is of great importance to many real-world applications, such as natural language processing, knowledge graph construction, and question answering. In particular, extracting entities and relations from the massive instructed texts has attracted increasing attention, and many efficient approaches have been proposed. To extract both entities and relations, early works adopt pipeline methods^1–3^. These methods first recognize entities and then predict the relationship between entities. However, pipeline methods do not consider the correlations between operations of entity recognition and relation extraction.

Recent advances in deep learning enable promising results in feature learning for relation extraction^4,5^. Existing deep learning approaches usually use convolution neural network (CNN) and recurrent neural network (RNN) or its variants long short-term memory (LSTM) for feature extraction. In particular, Zeng et al.^6^ use CNN for relation classification. Xu et al.^7^ propose RNN based relation classification model. Li et al.^4^ propose a fast relation extraction model with a convolutions encoder and adaptive threshold based on cascade binary tagging. Fu et al.^8^ employ graph convolution network (GCN) and bi-directional long short-term memory (Bi-LSTM) to jointly extract entities and relations. Zheng et al.^9^ design a potential relation prediction component and a relation-specific sequence tagging component to jointly extract relational triples and solve the overlapping problem.

Recent studies begin to explore the overlapping relation extraction problem and propose efficient solutions. Wei et al.^10^ propose a novel cascade binary tagging framework that models relations as functions to handle the overlapping problem. Li et al.^4^ design position-dependent adaptive threshold to improve the cascade binary tagging. Although deep learning based relation extraction has achieved excellent performance. However, there are still some problems that need to further explore:

- Existing improvements in relation extraction performance mainly rely on pre-trained language models such as bi-directional encoder representations from transformer (BERT)^11^, which is at the price of much time cost and memory consumption within GPUs. Designing a computation efficient solution for entity and relation extraction is meaningful.
- There are some overlapped entity pairs in some triplets. The models focus on the case that none of the triplets have overlapped entities and could not obtain satisfactory results.

To address the above problems, we propose a Hybrid Attention and Dilated convolution Framework (HADNet) in this paper. HADNet designs a novel encoder architecture that combines self-attention and a multi-scale extraction module with dilated convolution and a gated unit. HADNet has the advantage of computation efficiency. It enables us to enjoy global receptive fields meanwhile utilize the local context, consequently makes accurate entity and relation extraction. The contributions of this work are summarized as follows.

1. We propose a self-attention entity and relation extraction model-HADNet, which captures the features in sentences and provides better performance on entity and relation extraction.
2. We design a context-aware self-attention module as an encoder, which integrates multi-scale extraction with self-attention. The model enables self-attention to be aware of the local context.
3. We decompose the decoder into three phases, relation prediction, entity recognition, and relation determination, which avoids the redundancy of the relation extraction operation.
4. We conduct extensive experiments on real-world datasets to validate the performance of our proposed model. The experimental results show that our model can solve the overlapping triples problem in relational triple extraction and achieves competitive performances compared to the existing baselines.

The rest of the paper is organized as follows: Section “Related work” gives the related work; the proposed model is introduced in Section “Model training”, and experimental evaluations are presented in Section “Experimental studies”; Section “Conclusion” concludes the paper.

## Related work

Entity and relation extraction is a fundamental problem in knowledge construction and has attracted extensive research attention during the past decades. Earlier work is usually based on the pipeline^1–3^. For example, Zelenko et al.^1^ propose to use the devised kernels in conjunction with Support Vector Machine and Voted Perceptron learning algorithms to extract person-affiliation and organization-location relations from text. Chan et al.^3^ propose an algorithm that first identifies structures in the text and then identifies the semantic type of the relation with the extracted structures. However, pipeline methods do not consider the correlations between operations of entity recolonization and relation extraction.

Recently, deep learning has proven very effective in feature extraction and representation learning^4,12,13^. Many deep learning based approaches for entity and relation extraction have been proposed. Existing deep learning approaches usually adopt a CNN to encode sentence semantics, RNN, and its variant LSTM to model the temporal correlation of words in the sentence. In particular, Zeng et al.^6^ use CNN for relation classification. Xu et al.^7^ employ RNN for relation classification. Lin et al.^14^ propose a sentence-level attention method to make full use of the related information in all sentences and calculate the weighted sum of all sentences. Guo et al.^15^ introduce an entity recognition function to further obtain entity background knowledge and improve relation extraction performance. Xiao et al.^16^ propose a hybrid deep neural network model to jointly extract the entities and relations, moreover, the model is capable of filtering noisy data caused by distant supervision. Attention mechanism and graph neural network have been popular in recent years^17–19^. Xiao et al.^20^ propose an attention-based transformer block model for distant supervision relation extraction. The model could achieve richer vector expressions for each sentence and better address the wrong labeling problem. Zheng et al.^21^ propose a weighted relative position transformer encoder to capture the semantic relationship between entities flexibly.

Advancement in relation extraction enable applying it in many domains, such as recommendation system, and finding potential therapeutic targets in diseases^19,22,23^, etc. However, due to the influence of the structure, these approaches may not obtain satisfactory results. For example, CNN cannot encode temporal information between words in each pair of sentences, while RNN greatly prolongs the training time of the model because words need to be added to the calculation in the sequence, which makes it very difficult to encode long and complex sentences^24^. Moreover, RNN based method lacks fine-grained feature extraction.

Most of the existing works focus on relational triple extraction of sentences containing single triples, while few methods consider the problem of overlapping triples in the same sentence. Due to the complexity of languages, there may be more than one pair of entity pairs and relational triples in a single text, which means that there are multiple triples in a sentence. Zeng et al.^25^ propose the concept of overlapping triples and design a sequence-to-sequence model with copy mechanism. Zheng et al.^26^ propose to directly model triples as a whole and solve the entity and relation extraction problem. However, relational triples are still regarded as discrete labels, which results in excessive negative cases in model training and influence the extraction performance.

However, Transformer based pre-trained language models such as BERT consume much computation resources and memory in GPUs, which influences the training efficiency. Furthermore, existing two stages based relation extraction usually applies extraction operation to all relations, which results in much redundancy.

## Methodology

**Figure 1:**
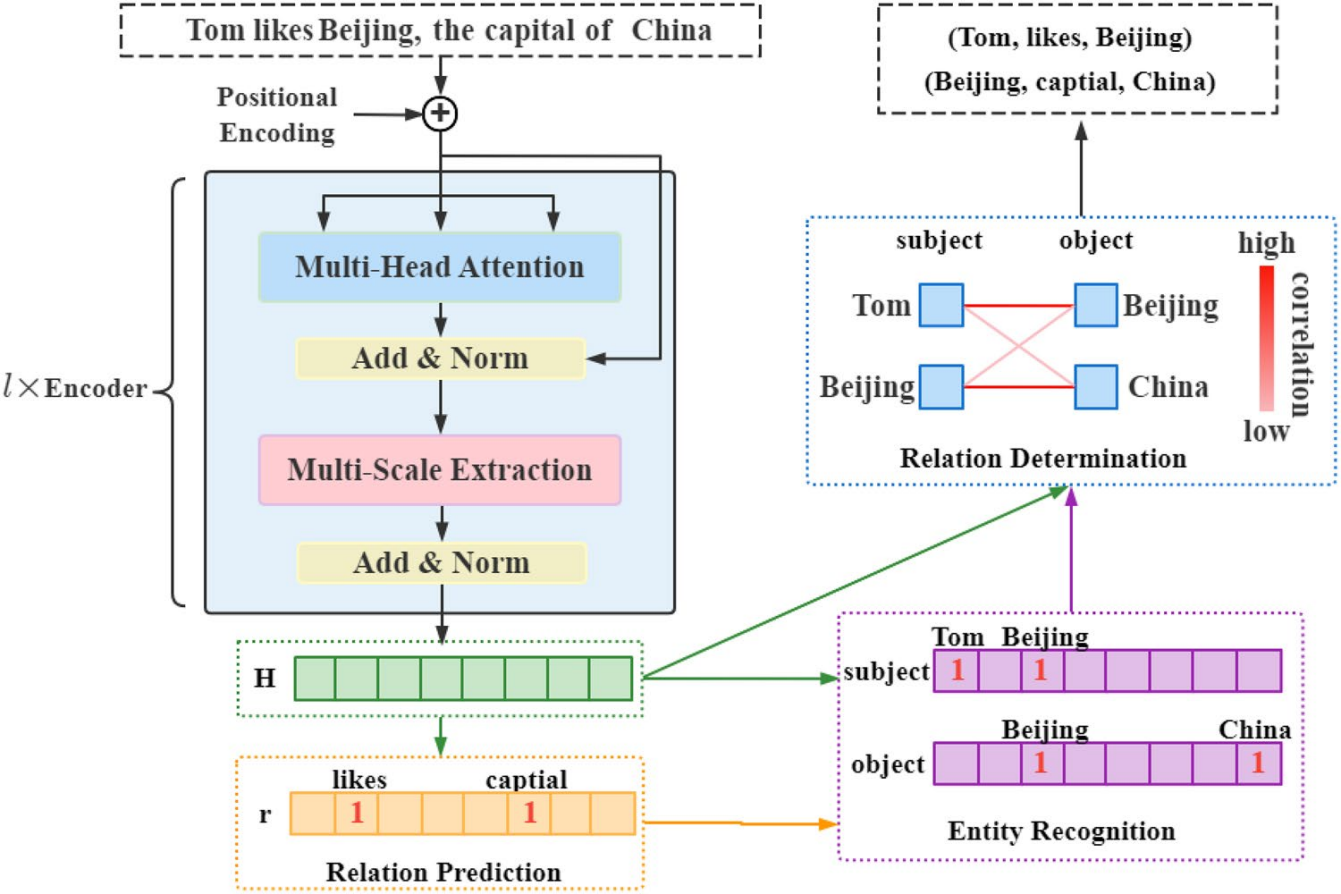
The architecture of HADNet. HADNet follows an encoder-decoder structure. The encoder stacks multiple self-attention blocks (i.e., blue blocks) and multi-scale extraction blocks (i.e, pink blocks). Given a sentence, the output of the encoder is the embedding *H*, which is fed into the HADNet decoder. The decoder is decomposed into three components, the relation prediction component generates potential relations. Based on this, the entity recognition component tags subjects and objects. Finally, the relation determination component remains the corrected subjects and object pairs.

The overall architecture of the proposed model is shown in Fig. 1, which adopts attention based Encoder-Decoder architecture. It first encodes a sentence into a fixed-length vector representation with an attention mechanism. After that, entity and relation extraction are conducted in the decoder.

### HADNet encoder

Self-Attention is efficient to process natural language data. To further improve the performance of entity and relation extraction, the encoder integrates self-attention with a multi-scale extraction module (MSE) to achieve a global receptive field and utilizes local context. Figure 1 presents the overall framework of the encoder, which is composed of *L* context-aware self-attention blocks. In each block, the multi-head attention focuses on the global features extraction, while MSE adopts dilated convolutions with the gated unit to capture local features.

Given the sentence, the output of the encoder is hidden states *H*. The details of the components of the encoder are described in the remaining subsections.

#### Multi-head attention

Multi-head attention is widely used in many self-attention mechanism based applications^27^. It aims to aggregate the information of the previous layer, which first maps the queries, keys, and values into three representation subspaces, namely *Q*, *K*, and *V* through *m* different linear transformations. Then, the attention function is performed in parallel^27^:

1 $$\begin{aligned} {Attention}(Q, K, V)={softmax}\left( \frac{Q K^{T}}{\sqrt{d_{k}}}\right) V \end{aligned}$$

where $d_k$ is the dimension of keys and values. Finally, the outputs are concatenated and further projected to obtain the final output:

2 $$\begin{aligned} head_i= & {} { Attention }\left( Q W_{i}^{Q}, K W_{i}^{K}, V W_{i}^{V}\right) \end{aligned}$$

3 $$\begin{aligned} {MHAttention}(Q, K, V)= & {} { Concat }\left( { head }_{1}, { head }_{2}, \ldots , { head }_{m}\right) W^{o} \end{aligned}$$

where *m* is the number of attention heads. $W_{i}^{Q}$, $W_{i}^{K}$ and $W_{i}^{V}$ are projection matrices used on *Q*, *K*, and *V*. $W^O$ is the final output projection matrix. The multi-head attention is efficient to capture the global features as it models the correlations of elements in sentences without considering their distance. However, the multi-head ignores the local trend information inherent in the sentence. To address the above problem, we further add a multi-scale extraction module, which considers the local contextual information.

#### Multi-scale extraction module

**Figure 2:**
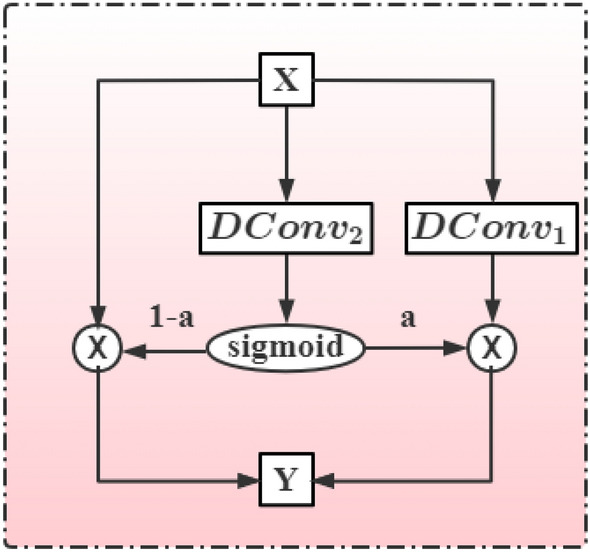
The architecture of Multi-Scale Extraction module (MSE).

To enhance the fine-grained coding ability of the model and capture more accurate correlations, we design the multi-scale extraction module (MSE). MSE can capture multi-scale local information in sentences explicitly, as shown in Fig. 2. The MSE applies dilated convolutions with gated units to exploit features in different scales of receptive fields^28^. In particular, we utilize two dilated convolutions at each layer to transform the input feature. After that, the features learned from different scales are fused employing residuals and gated unit to achieve a multi-scale representation^28^, which is denoted as:

4 $$\begin{aligned} \begin{aligned} a&=sigmoid(DConv_2(X))\\ Y&=X \otimes (1-a)+a\otimes DConv_1(X) \end{aligned} \end{aligned}$$

### HADNet decoder

In this section, we describe the design of HADNet decoder that consist of three components.

#### Relation prediction

The relation prediction component aims to predict a subset of relations that possibly exist in the sentence. Given the embedding $H=\{h_1,h_2,\ldots ,h_m\}$, the relation prediction function is formulated as:

5 $$\begin{aligned} P_{r}^{H}={sigmoid}\left( W_{r} H+b_{r}\right) \end{aligned}$$

where $W_r$ is the trainable weight, $b_r$ is the bias vector. Inspired by the cascade tagging method^10^, we further tag the relation with a threshold $T_r$. As shown in Fig. 1 , the probability tagger is set to 1 if its value is higher than $T_r$ or set to 0 if its value is lower than $T_r$.

#### Entity recognition

The entity recognition component aims to extract subjects and objects as the sequence tagging task. Let $h_i$ denote the representation of the *i*-th token,

6 $$\begin{aligned} P_ {i,j}^{s}= & {} sigmoid \left( W_{s}\left( h_{i}+r_{j}\right) +b_{s}\right) \end{aligned}$$

7 $$\begin{aligned} P_{i,j}^{o}= & {} sigmoid \left( W_{o}\left( h_{i}+r_{j}\right) +b_{o}\right) \end{aligned}$$

where $r_j$ is the *j*-th relation representation, $W_{s}$ and $W_{o}$ are trainable weights.

#### Relation determination

In the previous subsection, we have obtained possible subjects and objects according to their potential relations in the sentence. Next, we will capture the inter-dependencies between the subject and object pairs. Let $h_i^s$ and $h_j^o$ respectively denote the $i$-th token and $j$-th token in the sentence, and they form potential subject and object pair, cosine similarity between two entities is used as aggregated weights^29^,

8 $$\begin{gathered} P_{{ij}} = \rm{cosine}(h_{i}^{s} ,h_{j}^{o} ) \cdot w_{{ij}} = \frac{{h_{i}^{s} \times (h_{j}^{o} )^{T} \cdot w_{{ij}} }}{{\left\| {h_{i}^{s} } \right\| \times \left\| {h_{j}^{o} } \right\|}} \hfill \\ \hat{P}_{{ij}} = sigmoid(P_{{ij}} ) \hfill \\ \end{gathered}$$

where $w_{ij}$ is the weight matrix. Next, we determine the relation by comparing it with a threshold $T_d$. As shown in Fig. 1, if $\hat{P_{ij}}$ is higher the $T_d$, then the corresponding subject and object pair will be remained or be removed if the value is lower than $T_d$.

## Model training

The loss function is composed of three parts as follows:

9 $$\begin{aligned} {\varvec{L}}= {\varvec{L}}_{rp}+{\varvec{L}}_e+{\varvec{L}}_{rd} \end{aligned}$$

where ${\varvec{L}}_{rp}$, ${\varvec{L}}_e$, and ${\varvec{L}}_{rd}$ are the loss of relation prediction component, entity recognition component, and relation determination component, respectively, which are obtained via taking the log of the probabilities.

## Experimental studies

In this section, we report the experimental results of the proposed HADNet. We first introduce the Datasets, the experimental setting and the baselines. After that, we present the experimental results and evaluation discussions.

### Datasets

To test the performance of our proposed model, we use two public real-world datasets WebNLG^30^ and New York Times (NYT)^31^.

- NYT: It contains 24 predefined relation types, the dataset consists of 1.18M sentences of news articles from 1987-2007 New York Times. And it is produced by the distant supervision method. We follow the existing work’s preprocessing steps^10^ to split the dataset, sentences for training, validation, and test are 56195, 5000 and 5000 respectively.
- WebNLG: It is created for natural language processing tasks, and Zeng et al.^25^ first utilizes it for relation extraction tasks. The dataset contains 246 valid relations. We follow Wei et al.^10^’s preprocessing steps to split the dataset, sentences for training, validation and test are 5019, 500 and 703 respectively.

### Baselines and evaluation metrics

We compare HADNet with the following widely used baselines. All the experimental results of the baseline methods are directly obtained from^10^ unless specified.

- NovelTagging^26^: Sequence annotation relational triple extraction based on entity relation joint decoding.
- CopyR$_{OneDecoder}$^25^: End-to-end relational extraction model based on a single decoder.
- CopyR$_{MultiDecoder}$^25^: End-to-end relational triple extraction model based on multiple decoders.
- GraphRel$_{1p}$^8^: Relational triple extraction model based on graph convolutional neural network.
- GraphRel$_{2p}$^8^: Graph convolutional neural network model for relational triple extraction based on fusing relation weighted vector.
- CopyR$_{RL}$^32^: Relational triple extraction model based on reinforcement learning.
- CASREL$_{random}$^10^: Cascade binary tagging framework when all parameters of BERT are randomly initialized.

Following Zheng et al.^26^, the performance of different models is evaluated with the following metrics: precision, recall, and F1 scores.

### Experimental results

Tables 1 and 2 show the precision, recall, and F1 scores of our proposed model as compared to other baselines on WebNLG and NYT datasets, respectively. From the tables, we can draw the following conclusions: (1) Our HADNet model outperforms the state-of-the-art models not based on BERT. Only in the WebNLG dataset, recall score of HADNet is slightly lower than that of the CopyR${_{RL}}$ model, while both precision score and F1 score are higher than that of the CopyR${_{RL}}$ model. Besides, there are 12 and 3% improvements in F1 values over the two datasets, respectively. (2) While comparing with model based on BERT (CASREL$_{random}$), in the WebNLG dataset, our model obtains the best precision score, which is 88.8. There is 3% improvement compared with the CASREL$_{random}$ model, which is 84.7. In the NYT dataset, the precision score of our model is 81.2, which is competitive with the precision score of the CASREL$_{random}$ model (81.5). These facts imply the effectiveness of our models.

**Figure 3:**
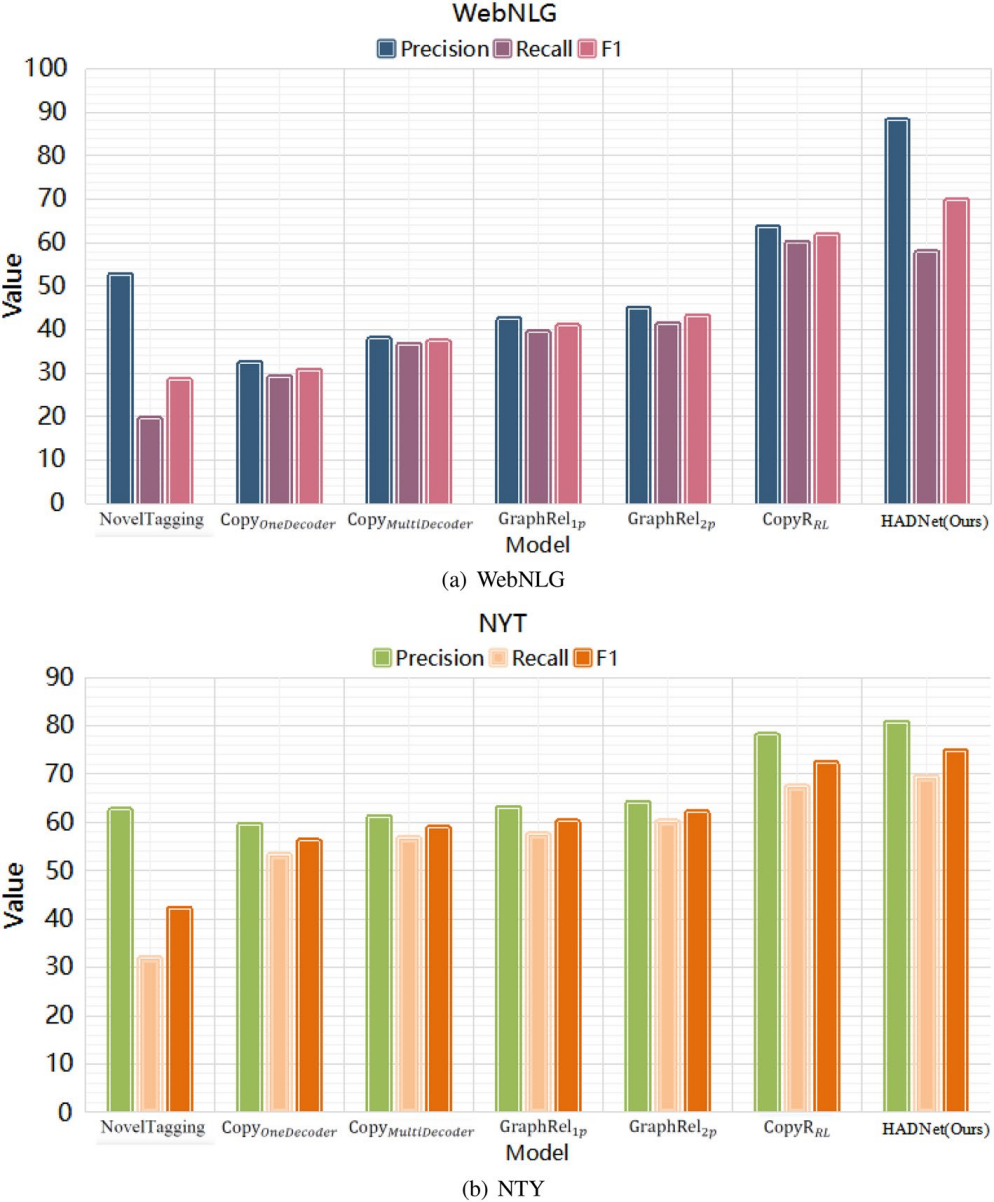
Results of different models (without BERT) over WebNLG and NYT datasets.

**Figure 4:**
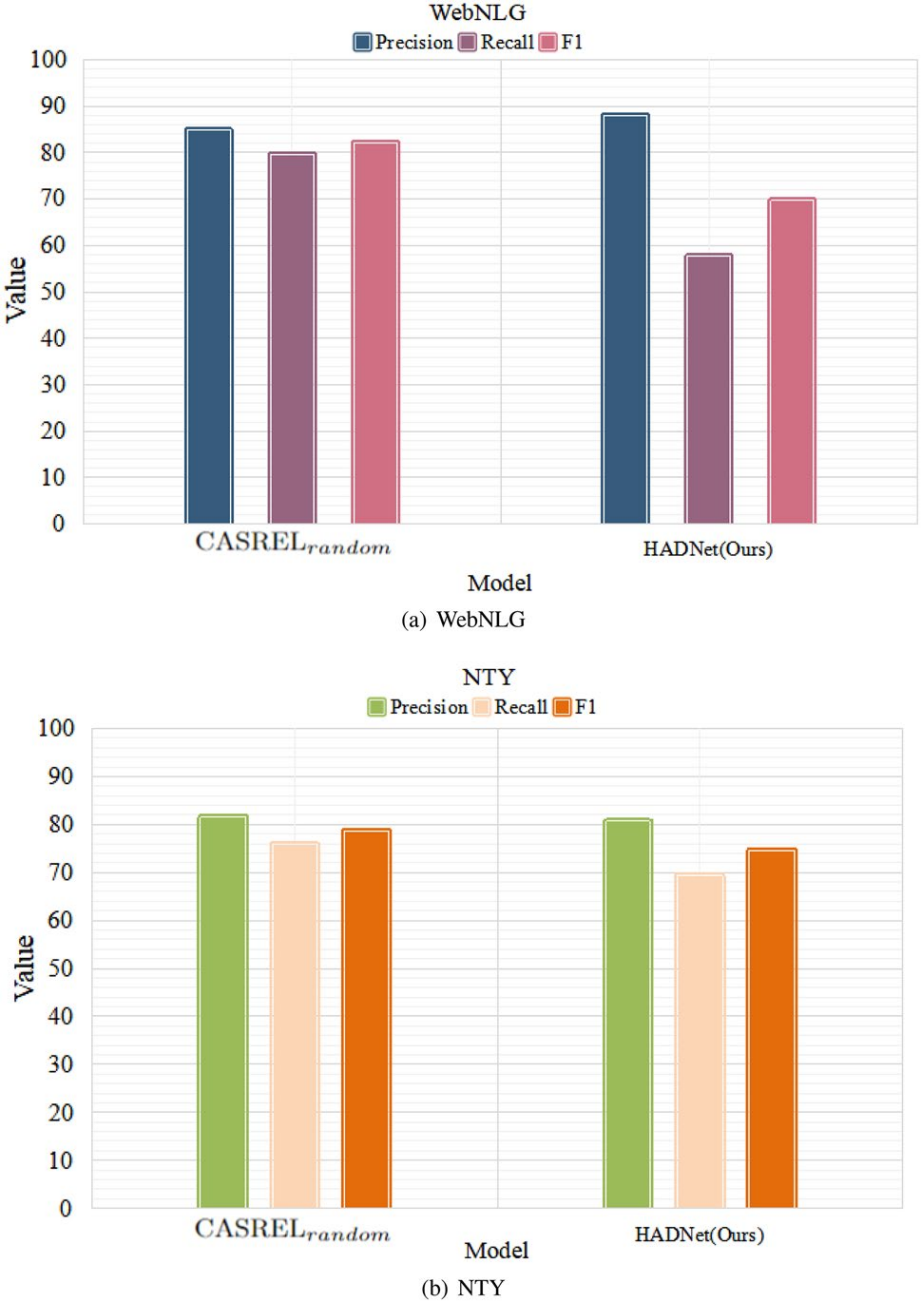
Results of HADNet and CASREL (with BERT) over WebNLG and NYT datasets.

Figures 3 and 4 show the performance of different models under different evaluation metrics over the two datasets. We can see that our model has better performance than other models not using BERT as the pr-trained model. It is also observed that the performance on NYT of HADNet is not good as the model based on the BERT. The reason is that HADNet adopts a simple and efficient mechanism to approximate pre-trained model BERT, which results in limited representation ability. Nonetheless, HADNet still outperforms models not based on BERT such as CopyR$_{RL}$, GraphRel, and close to CASREL$_{random}$. It implies that self-attention and dilated convolution based model is able to achieve stable and competitive expression ability.

**Table 1:** Results of different models (without BERT) over WebNLG and NYT datasets.

Baseline methods	WebNLG			NYT		
	Precision	Recall	F1	Precision	Recall	F1
NovelTagging	52.5	19.3	28.3	62.4	31.7	42.0
CopyR$_{OneDecoder}$	32.2	28.9	30.5	59.4	53.1	56.0
CopyR$_{MultiDecoder}$	37.7	36.4	37.1	61.0	56.6	58.7
GraphRel$_{1p}$	42.3	39.2	40.7	62.9	57.3	60.0
GraphRel$_{2p}$	44.7	41.1	42.9	63.9	60.0	61.9
CopyR$_{RL}$	63.3	**59.9**	61.6	77.9	67.2	72.1
HADNet(Ours)	**88.8**	57.1	**69.2**	**81.2**	**70.1**	**74.8**

The [bold] values is the the best result after comparing each method.

**Table 2:** Results of HADNet and CASREL (with BERT) over WebNLG and NYT dataset.

Baseline methods	WebNLG			NYT		
	Precision	Recall	F1	Precision	Recall	F1
CASREL$_{random}$	84.7	**79.5**	**82**	**81.5**	**75.7**	**78.5**
HADNet(Ours)	**88.8**	57.1	69.2	81.2	70.1	74.8

The [bold] values is the the best result after comparing each method.

Following previous works^10,33,34^, we further conduct experiments over the NYT dataset to explore the performance of HADNet for solving overlapping problems, and the results are shown in Table 3. We can see that our model obtains satisfactory performance under different overlapping patterns. Moreover, the performance even improves under the EPO and SEO patterns. It implies that our model is competitive in solving the extraction task of overlapping triples.

**Table 3:** Results of different overlapping patterns over the NYT dataset.

Pattern	NYT		
	Precision	Recall	F1
Normal	77.8	71.0	72.9
EPO	83.5	79.1	81.2
SEO	80.3	73.9	76.8

**Figure 5:**
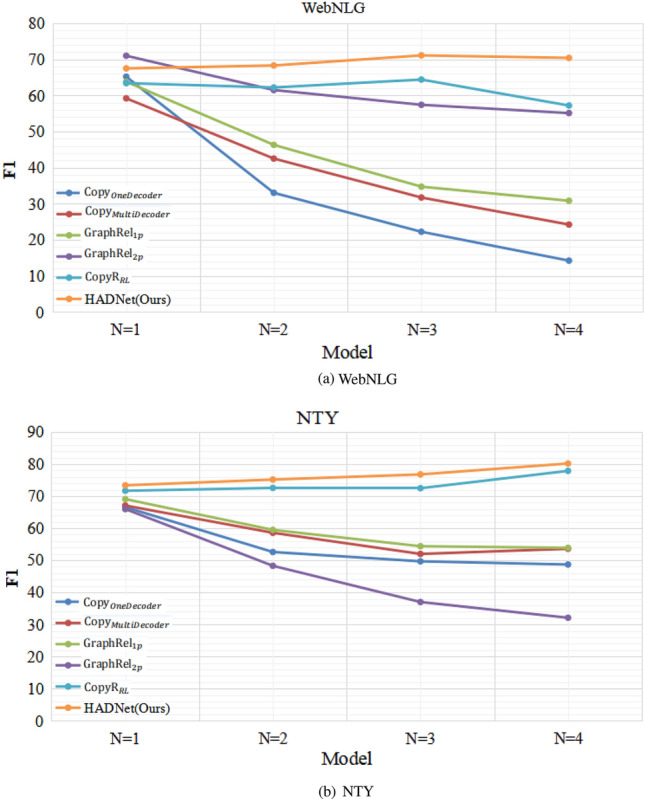
F1-score of sentences with different numbers of triples.

Table 4 shows the F1-score of sentences with different numbers of triples, where *N* is the number of triples in a sentence. Compared with the baseline models, HADNet achieves excellent results when *N* varies from 1 to 4. From Fig. 5, we can see that the performance of all models is the best when $N=1$, while the performance declines considerably with the increasing of *N*. Although our model declines with the increasing of *N* as well, it is still better than other baselines and obtains consistently better performance with different *N*.

**Table 4:** F1-score of sentences with different numbers of triples.

Baseline models	NYT				WebNLG			
	$N=1$	$N=2$	$N=3$	$N=4$	$N=1$	$N=2$	$N=3$	$N=4$
CopyR$_{OneDecoder}$	66.6	52.6	49.7	48.7	65.2	33.0	22.2	14.2
CopyR$_{MultiDecoder}$	67.1	58.6	52.0	53.6	59.2	42.5	31.7	24.2
GraphRel$_{1p}$	69.1	59.5	54.4	53.9	63.8	46.3	34.7	30.8
GraphRel$_{2p}$	71.0	61.5	57.4	55.1	66.0	48.3	37.0	32.1
CopyR$_{RL}$	71.7	72.6	72.5	77.9	63.4	62.2	64.4	57.2
HADNet(Ours)	73.6	74.1	75.9	81.1	68.1	68.9	70.9	69.9

## Conclusion

Entity and relation extraction have attracted continuous attentions in recent years. However, the overlapping triples problem and training efficiency propose challenges for it. To tackle these problems, in this paper, we proposed a novel Hybrid Attention and Dilated convolution Network (HADNet), which considered the computation efficiency and overlapping triples while maintaining competitive performance. In particular, We first designed a novel encoder that combined self-attention with dilated convolution and a gated unit for efficient relation extraction. Then, we employed cosine similarity schemes to determine relations. Finally, when evaluated on two real-world datasets, the proposed model achieved better results than state-of-the-art baselines that do not use BERT as a pre-trained model. For our future work, we intend to explore jointly learns named entities and relations based on graph convolutional networks. Moreover, we plan to conduct the proposed model on more datasets to verify the universality and effectiveness of our method.

## Data Availability

The datasets used and/or analysed during the current study available from the corresponding author on reasonable request.

## References

[1] Zelenko D, Aone C, Richardella A (2003). Kernel methods for relation extraction. J. Mach. Learn. Res..

[2] Zhou, G., Su, J., Zhang, J. & Min, Z. Exploring various knowledge in relation extraction. In *Acl, Meeting of the Association for Computational Linguistics, Conference, June, University of Michigan, USA* 1–8 (2002).

[3] Chan, Y. S. & Dan, R. Exploiting syntactico-semantic structures for relation extraction. In *The 49th Annual Meeting of the Association for Computational Linguistics: Human Language Technologies, Proceedings of the Conference, 19–24 June, 2011, Portland, Oregon, USA* 551–560 (2011).

[4] Li, G., Chen, X., Wang, P., Xie, J. & Luo, Q. Fastre: Towards fast relation extraction with convolutional encoder and improved cascade binary tagging framework. In *Proceedings of the Thirty-First International Joint Conference on Artificial Intelligence, IJCAI 2022, Vienna, Austria, 23–29 July 2022* (ed. Raedt, L. D.) 4201–4208, 10.24963/ijcai.2022/583 (2022).

[5] Li Z, Ren Q, Chen L, Li J, Li X (2022). Multi-scale convolutional networks for traffic forecasting with spatial-temporal attention. Pattern Recognit. Lett..

[6] Zeng, D., Liu, K., Lai, S., Zhou, G. & Zhao, J. Relation classification via convolutional deep neural network. In *COLING 2014, 25th International Conference on Computational Linguistics, Proceedings of the Conference: Technical Papers, 23–29 August, 2014, Dublin, Ireland* (eds. Hajic, J. & Tsujii, J.) 2335–2344 (2014).

[7] Xu, Y. *et al.* Classifying relations via long short term memory networks along shortest dependency paths. In *Proceedings of the 2015 Conference on Empirical Methods in Natural Language Processing, EMNLP 2015, Lisbon, Portugal, 17–21 September, 2015* 1785–1794 (2015).

[8] Fu, T., Li, P. & Ma, W. Graphrel: Modeling text as relational graphs for joint entity and relation extraction. In *Proceedings of the 57th Conference of the Association for Computational Linguistics, ACL 2019, Florence, Italy, 28 July–2 August, 2019, Volume 1: Long Papers* 1409–1418. 10.18653/v1/p19-1136 (2019).

[9] Zheng, H. *et al.* PRGC: Potential relation and global correspondence based joint relational triple extraction. In *Proceedings of the 59th Annual Meeting of the Association for Computational Linguistics* 6225–6235. 10.18653/v1/2021.acl-long.486 (2021).

[10] Wei, Z., Su, J., Wang, Y., Tian, Y. & Chang, Y. A novel cascade binary tagging framework for relational triple extraction. In *Proceedings of the 58th Annual Meeting of the Association for Computational Linguistics* 1–13 (2020).

[11] Devlin, J., Chang, M. W., Lee, K. & Toutanova, K. Bert: Pre-training of deep bidirectional transformers for language understanding. arXiv preprint arXiv:1810.04805 (2019).

[12] Zhou, Y. *et al.* Abnormal activity detection in edge computing: A transfer learning approach. In *2020 International Conference on Computing, Networking and Communications (ICNC)* 107–111 (IEEE, 2020).

[13] Wang W, Zhang L, Sun J, Zhao Q, Shuai J (2022). Predicting the potential human lncRNA–miRNA interactions based on graph convolution network with conditional random field. Brief Bioinform..

[14] Lin, Y., Shen, S., Liu, Z., Luan, H. & Sun, M. Neural relation extraction with selective attention over instances. In *Proceedings of the 54th Annual Meeting of the Association for Computational Linguistics, ACL 2016, 7–12 August, 2016, Berlin, Germany, Volume 1: Long Papers* 2124–2133. 10.18653/v1/p16-1200 (2016).

[15] Ji, G., Liu, K., He, S. & Zhao, J. Distant supervision for relation extraction with sentence-level attention and entity descriptions. In *Proceedings of the Thirty-First AAAI Conference on Artificial Intelligence, 4–9 February, 2017, San Francisco, California, USA* (eds. Singh, S. & Markovitch, S.) 3060–3066 (2017).

[16] Xiao, Y., Tan, C., Fan, Z., Xu, Q. & Zhu, W. Joint entity and relation extraction with a hybrid transformer and reinforcement learning based model. In *National Conference on Artificial Intelligence* 9314–9321 (2020).

[17] Wang T, Sun J, Zhao Q (2023). Investigating cardiotoxicity related with hERG channel blockers using molecular fingerprints and graph attention mechanism. Comput. Biol. Med..

[18] Ren Q, Li Y, Liu Y (2023). Transformer-enhanced periodic temporal convolution network for long short-term traffic flow forecasting. Expert Syst. Appl..

[19] Sun F, Sun J, Zhao Q (2022). A deep learning method for predicting metabolite-disease associations via graph neural network. Brief Bioinform..

[20] Xiao Y, Jin Y, Cheng R, Hao K (2022). Hybrid attention-based transformer block model for distant supervision relation extraction. Neurocomputing.

[21] Zheng W, Wang Z, Yao Q, Li X (2021). WRTRe: Weighted relative position transformer for joint entity and relation extraction. Neurocomputing.

[22] Li, X. *et al.* Caspase-1 and Gasdermin D afford the optimal targets with distinct switching strategies in nlrp1b inflammasome-induced cell death. In *Research (Wash D C)* 1–17 (2022). 10.34133/2022/9838341 PMC9343085 35958114

[23] Tian Y, Li G, Sun P (2021). Bridging the information and dynamics attributes of neural activities. Phys. Rev. Res..

[24] Liu, L., Priestley, J. L., Zhou, Y., Ray, H. E. & Han, M. A2text-net: A novel deep neural network for sarcasm detection. In *IEEE International Conference on Cognitive Machine Intelligence* 118–126 (2019).

[25] Zeng, X., Zeng, D., He, S., Liu, K. & Zhao, J. Extracting relational facts by an end-to-end neural model with copy mechanism. In *Proceedings of the 56th Annual Meeting of the Association for Computational Linguistics, ACL 2018, Melbourne, Australia, 15–20 July, 2018, Volume 1: Long Papers* (eds. Gurevych, I. & Miyao, Y.) 506–514 (2018).

[26] Zheng, S. *et al.* Joint extraction of entities and relations based on a novel tagging scheme. In *Proceedings of the 55th Annual Meeting of the Association for Computational Linguistics, ACL 2017, Vancouver, Canada, 30 July–4August 4, Volume 1: Long Papers* (eds. Barzilay, R. & Kan, M.) 1227–1236 (2017).

[27] Vaswani, A. *et al.* Attention is all you need. arXiv (2017).

[28] Yu, F. & Koltun, V. Multi-scale context aggregation by dilated convolutions. In *ICLR* 1–13 (2016).

[29] Wang Y, Ren Q, Li J (2023). Spatial-temporal multi-feature fusion network for long short-term traffic prediction. Expert Syst. Appl..

[30] Gardent, C., Shimorina, A., Narayan, S. & Perez, L. Creating training corpora for micro-planners. In *In Proceedings of the 55th Annual Meeting of the Association for Computational Linguistics* 367–377 (2017).

[31] Riedel S, Yao L, Mccallum AK (2010). Modeling Relations and Their Mentions Without Labeled Text.

[32] Zeng, X. *et al.* Learning the extraction order of multiple relational facts in a sentence with reinforcement learning. In *Empirical Methods in Natural Language Processing* 367–377 (2019).

[33] Wang, Y. *et al.* Tplinker: Single-stage joint extraction of entities and relations through token pair linking. In *Proceedings of the 28th International Conference on Computational Linguistics, COLING 2020, Barcelona, Spain (Online), 8–13 December, 2020* (eds. Scott, D., Bel, N. & Zong, C.) 1572–1582 (2020).

[34] Yuan, Y. *et al.* A relation-specific attention network for joint entity and relation extraction. In *Proceedings of the Twenty-Ninth International Joint Conference on Artificial Intelligence, IJCAI 2020* (ed. Bessiere, C.) 4054–4060 (2020).

